# Comparison of endoscopic ultrasound-guided hepaticogastrostomy and the antegrade technique in the management of unresectable malignant biliary obstruction: study protocol for a prospective, multicentre, randomised controlled trial

**DOI:** 10.1186/s13063-020-04758-5

**Published:** 2020-09-29

**Authors:** Ye Liao, Marc Giovannini, Ning Zhong, Tingyue Xiao, Shiyun Sheng, Yufan Wu, Jingjing Zhang, Sheng Wang, Xiang Liu, Siyu Sun, Jintao Guo

**Affiliations:** 1 Department of gastroenterology, Shengjing Hospital of China Medical University, No. 36, Sanhao Street, Shenyang, 110004 China; 2 Endoscopy Unit, Paoli Calmettes Institute, 232 bd Ste Marguerite, 13009 Marseille, France; 3 Department of Gastroenterology, Qilu Hospital, Cheeloo College of Medicine, Shandong University, Jinan, 250012 China; 4 The Sixth People’s Hospital of Shenyang, Shenyang, 110006 China

**Keywords:** Intrahepatic EUS-BD, Malignant biliary obstruction, EUS-HGS, EUS-AG, Randomised controlled trial

## Abstract

**Background:**

Endoscopic ultrasound-guided biliary drainage (EUS-BD) is used after failed endoscopic retrograde cholangiopancreatography. Based on existing studies, intrahepatic (IH) approaches are preferred in patients with dilated IH bile ducts. Both ultrasound-guided hepaticogastrostomy (EUS-HGS) and ultrasound-guided antegrade treatment (EUS-AG) are appropriate for patients with unreachable papillae. Nevertheless, there have been no direct comparisons between these two approaches. Therefore, we aim to evaluate and compare the safety and efficiency of EUS-HGS and EUS-AG in patients with an unreachable papilla.

**Methods:**

This is a prospective, randomised, controlled, multicentre study with two parallel groups without masking. One hundred forty-eight patients from three hospitals who met the inclusion criteria will be randomly assigned (1:1) to undergo either EUS-HGS or EUS-AG for relief of malignant biliary obstruction. The final study follow-up is scheduled at 1 year postoperatively. The primary endpoint is efficiency, described by technical and clinical success rates of EUS-HGS and EUS-AG in patients with unreachable papillae. The secondary endpoints include stent patency, overall survival rates, complication rates, length of hospital stays, and hospitalisation expenses. The chi-square test, Kaplan–Meier methods, log-rank test, and Cox regression analysis will be used to analyse the data.

**Discussion:**

To our knowledge, this is the first study to compare these two EUS-BD approaches directly using a multicentre, randomised, controlled trial design. The clinical economic indexes will also be compared, as they may also affect the patient’s choice. The result may contribute to establishing a strategic guideline for choosing IH EUS-BD approaches.

**Trial registration:**

Chinese Clinical Trial Registry (ChiCTR) ChiCTR1900020737. Registered on 15 January 2019

## Background

Obstructive jaundice is the main cause of death associated with malignant biliary tumours. However, because of its insidious onset, the detection of cholangiocarcinomas often occurs too late for surgical resection of the primary tumour. Endoscopic retrograde cholangiopancreatography (ERCP) has been the standard procedure for palliative biliary drainage in patients with both benign and malignant biliary obstructions [1]. Nevertheless, there remains a failure rate of ERCP because of the difficulty of cannulation occasioned by variations in ampullary anatomy [1–3]. The traditional way of relieving biliary obstruction after failed ERCP is percutaneous transhepatic biliary drainage. This long-term external drainage method can cause electrolyte imbalances, and the persistent pain and repeated infections associated with abdominal wall fistulas can severely affect the quality of life [3–6]. Therefore, since it was first described in 2001, ultrasound-guided biliary drainage (EUS-BD) has been increasingly used as an endoscopic alternative to failed ERCP because of its high success rate, low adverse event rate, and advantage of immediate internal drainage [7–9].

According to the drainage route, EUS-BD can be categorised into transduodenal extrahepatic approaches and transgastric intrahepatic (IH) approaches. With the advantage of a lower bile leakage rate and better retention of the original anatomical structure, the IH approaches are suggested to be the first choice for patients with a dilated IH bile duct after failed ERCP [10, 11]. IH approaches include the rendezvous technique (RV), ultrasound-guided hepaticogastrostomy (EUS-HGS), and ultrasound-guided antegrade treatment (EUS-AG). Because of the requirement of retrograde stent implantation, RV is not feasible for patients with inaccessible papillae, whereas hepaticogastrostomy (HGS) and the antegrade technique (AG) are [12, 13]. However, there is no consensus regarding options for biliary access [14, 15]. For patients with malignant biliary obstruction, it is a great challenge to tolerant procedures once and experience postoperative complications. Therefore, it is important to develop strategies for choosing among the approaches (EUS-HGS or EUS-AG) by comparing their safety and efficiency.

To date, several retrospective studies have compared EUS-HGS and EUS-AG; nevertheless, there has been no well-designed prospective, randomised study with robust data on this topic. Therefore, in the present study, we aim to compare the safety and efficiency of EUS-HGS and EUS-AG in patients with unreachable papillae using a prospective, multicentre, randomised, controlled trial.

## Methods/design

### Ethical statements

The study was approved by the Medical Scientific Research and New Technology Ethics Committee of Shengjing Hospital of China Medical University (approval number: 2018PS525K) on 22 November 2018. Subsequently, the boards of the two participating hospitals gave permission to conduct the trial. Informed consent will be obtained from each participant or from each of the participant’s legally responsible relative. The trial was registered in the Chinese Clinical Trial Registry (ChiCTR), ChiCTR1900020737, on 15 January 2019.

### Participating centres

The three participating centres, including Shengjing Hospital of China Medical University in China, Qilu Hospital of Shandong University in China, and Institut Paoli Calmettes in France, were selected at the workshop of the International Society of Endoscopic Ultrasound (ISEUS). This selection was based on the fact that a few experts are capable of performing EUS-AG over the world, and the number of EUS-BD procedures is relatively higher at these centres. The background characteristics and procedures of this protocol were approved by all the centres involved.

### Patients

**Fig. 1:**
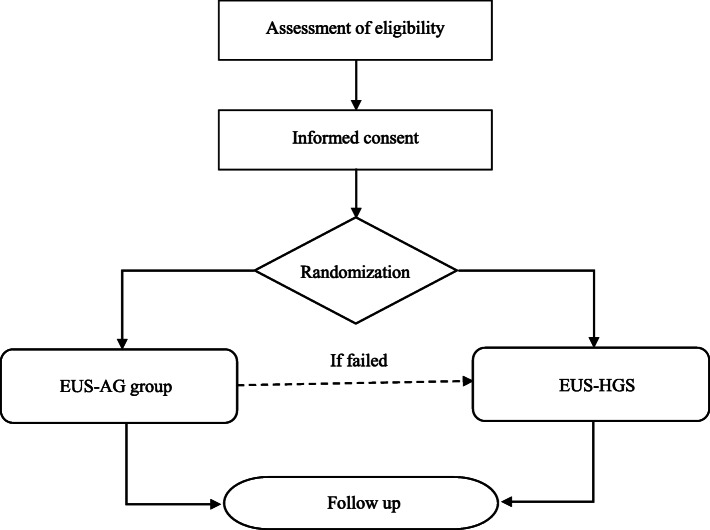
A flowchart of the study design

Patient recruitment will be carried out in three centres by gastroenterologists, surgeons, or endoscopists in their own centre who will evaluate the cases in the inpatient wards or in the outpatient consultation areas. After detailed introductions of this trial, patients will be given the opportunity to ask questions and decide whether to participate. The patients will also be reminded that consent is completely voluntary and can be withdrawn at any time. Patients who decide to participate will be randomly assigned (1:1) to receive EUS-HGS or EUS-AG for relief of biliary obstruction. A flowchart of the study design is shown in Fig. 1.

### Inclusion criteria

Patients with unresectable malignant biliary obstruction must meet the following inclusion criteria:

1. IH bile duct dilation confirmed by ultrasonography or CT
2. Failed ERCP
3. Anatomical abnormalities (congenital malformations, upper digestive tract surgery, or tumour mass)
4. Distal biliary obstruction and pyloric or duodenal obstruction caused by tumours
5. Informed consent

### Exclusion criteria

Patients with clear contraindications to endoscopy will be excluded:

1. Haemoglobin level ≤ 8.0 g/dl
2. Coagulopathy (platelet count < 50,000/mm^3^, international normalised ratio > 1.5) or having taken oral anticoagulation agents, such as aspirin or warfarin, in the previous week
3. Severe cardiorespiratory dysfunction
4. Psychiatric disease, drug addiction, or other reason for unreliable follow-up or responses to questionnaires
5. Other conditions that negatively affect compliance, which place the patient at increased risk, or otherwise make them unsuitable for participation

### Primary endpoints

The primary endpoint is the efficiency of HGS and AG described by technical success and clinical remission rates. Technical success is defined as successful stent placement which is clarified by endoscopists soon after the operation. Clinical remission will be evaluated 1 month postoperatively as it is defined as a decrease in the serum bilirubin level to less than 75% of preprocedural values within 30 days after stent placement. Only patients with both technical and clinical success are considered to reach the primary endpoint.

### Secondary endpoints

The secondary endpoints are as follows:

1. The overall survival rate for patients undergoing EUS-HGS and EUS-AG
2. Stent patency, complication rate, length of hospital stays, and hospitalisation expenses of EUS-HGS
3. Stent patency, complication rate, length of hospital stays, and hospitalisation expenses of EUS-AG

### Randomisation and interventions

The randomisation list will be generated by an independent statistician using block randomisation with variable block size (four or six) and kept in sealed envelopes without access by the investigators. Once a patient is enrolled by project secretary, the trial coordinator in Endoscopy Center of Shengjing Hospital of China Medical University will open an envelope and publish the final result of randomisation to all trial participants after confirmation, as this is an open-label design.

The equipment used will include a linear array echoendoscope (EG3830UT; Pentax, Tokyo, Japan) in combination with an ultrasound scanner (EUB 6500; Hitachi, Tokyo, Japan). A 19-G needle (EUS N-19-T; Wilson-Cook Medical, Winston-Salem, NC, USA) will be used for puncture, and a 0.035-in. guidewire (Jagwire; Boston Scientific, Natick, MA, USA) will be used for guidance. A cystotome (6 Fr; Wilson-Cook Medical) will be used to dilate the tract and create a large fistula. A fully covered metallic stent (Wilson-Cook Medical/Boston Scientific) or bare metallic stent (Wilson-Cook Medical/Boston Scientific) will be used for biliary drainage.

The former part of the procedure is the same for both EUS-AG and EUS-HGS. First, the echoendoscope will be advanced into the stomach, and the left lateral lobe liver will be scanned. A dilated IH bile duct close to the gastric wall will be selected as the puncture point. Then, to avoid the blood vessels in the puncture path, the local vasculature will be checked using colour Doppler ultrasonography, and a 19-G ultrasound puncture needle will be advanced into the IH duct. Next, bile aspiration and cholangiography will be performed to further clarify the location of the puncture needle and delineate the dilated biliary tree down to the point of obstruction.

In the AG group, a guidewire will be inserted through the puncture needle and guided into the intestine through the ampulla or anastomosis. A temporary fistula between the stomach (or jejunum in patients with total gastrectomy) and the left hepatic duct will be formed by cystectomy. Once the fistula has been dilated, a self-expandable bare metallic stent measuring 6–8 cm will be deployed into the malignant biliary obstruction in an antegrade fashion.

In the HGS group, a guidewire will be inserted through the needle and placed in the common bile duct. A cystotome will be used to create a fistula, and a fully covered metallic stent will be deployed into the fistula between the stomach (or jejunum in patients with total gastrectomy) and IH bile duct. Sometimes, a bare stent will be placed through the covered stent to avoid stent migration.

Finally, all devices will be removed after confirming with a contrast agent that bile flows well through the stent or stricture. To avoid bile leakage into the peritoneum, a 7-Fr naso-biliary catheter will sometimes be placed through the metallic stent for 48 h. In cases of failed EUS-AG, EUS-HGS will be performed for being recorded as technical failed cases in the EUS-AG group. To ensure data integrity, this group of patients will still be followed up as scheduled and recorded properly.

Both EUS-HGS and EUS-AG are established procedures of biliary drainage; there are no anticipated problems that would be detrimental to our patients. We do not anticipate the need for formal stopping rules for the trial; nevertheless, severe adverse events will be recorded in a timely manner and will be supervised by the monitoring board.

A Consolidated Standards of Reporting Trials (CONSORT) checklist for this study is provided in Additional file 1. The Standard Protocol Items: Recommendations for Interventional Trials (SPIRIT) checklist is provided in Additional file 2.

### Data collection

The baseline assessment will be performed after randomisation. Basic information including sex, age, race, and diagnosis will be recorded retrospectively. Concerning feasibility and safety, complete blood counts, hepatic and renal function tests, and biochemical tests will be performed on the day before the operation. Ultrasonography or upper abdominal computed tomography (CT) will be performed before the operation to confirm IH biliary dilation caused by obstruction.

Patients will undergo an examination of their bilirubin levels on days 1 and 3 postoperatively to evaluate the efficiency of the operations. Upper abdominal CT will be performed postoperatively to clarify the position of the stent. Technical success is defined as successful stent placement, and clinical remission is defined as a decrease in the serum bilirubin level to less than 75% of preprocedural values within 30 days after stent placement.

Adverse events at any time will be recorded and classified as post-procedure (up to 14 days) and late (any time after 14 days) and graded according to the American Society for Gastrointestinal Endoscopy lexicon’s severity grading system [16].

Stent patency is described by time to stent dysfunction needing re-intervention. The criteria for stent dysfunction are as follows: (1) cholangitis, (2) 50% increase in bilirubin from the lowest level post-index procedure, (3) 20% increase in bilirubin from the lowest level post-index procedure and evidence of obstruction on imaging, and (4) endoscopic or radiological re-intervention confirming stent blockage or migration.

**Fig. 2:**
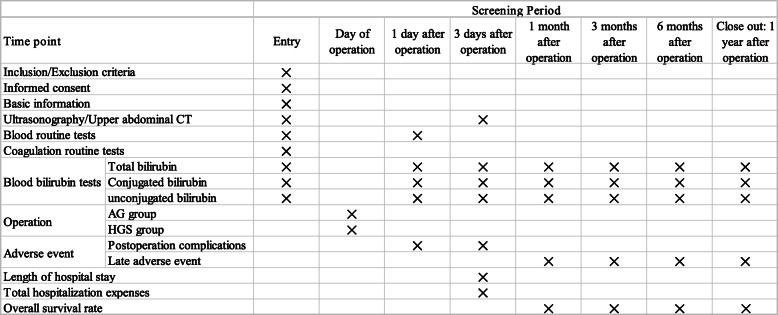
The schedule of enrolment, interventions, and assessments

Additionally, hospitalisation data including the length of hospital stay and total hospitalisation expenses will be recorded. The schedule of enrolment, interventions, and assessments is shown in Fig. 2.

### Follow-up

The final study follow-up is scheduled at 1 year postoperatively and includes evaluation of perioperative mortality and operative complications. Follow-up assessments of the bilirubin level (total bilirubin, conjugated bilirubin, and unconjugated bilirubin) and survival status are scheduled at 1 month, 3 months, 6 months, and 1 year after the operation or until death. If follow-up information is unable to be obtained, the patient will be considered lost to follow-up, and their data will be documented accordingly.

### Statistical analysis

#### Sample size calculation

The statistical significance level is set at 5%, power of the test is set as 80%, and randomisation ratio is set as 1:1. Based on previous data, the overall success rate of EUS-HGS is 84.5–100% with complication rates of 19.6–27% [5, 17–20]. The overall success and complication rates of EUS-AG are 57–100% and 0–5%, respectively [11, 21, 22]. Using a standard sample size formula, we calculated that 74 patients per group will be needed for a total of 148 patients, after accounting for a 10% dropout rate. A provisional deadline for patient recruitment is set in July 2022; however, in case the target number of patients has not been met, the recruitment period may be extended to reach the number required (2 × 67 patients) to obtain a power of at least 0.8 (80%).

#### Statistical analysis

The full analysis set (FAS) should be as close as possible to the intention-to-treat set. On the basis of the FAS, patients assigned to the two subgroups, including the HGS and AG groups, would form the per-protocol set. The direct deletion method will be used to treat missing data.

The technical success rate, clinical remission rate, and complication rate will be presented with 95% confidence intervals and compared between the procedures using the chi-square test. Normally distributed continuous variables, such as stent patency, bilirubin level, length of hospital stays, and hospitalisation expenses will be expressed as mean ± standard deviation and will be compared using the *t* test. Concerning the secondary endpoint, Kaplan–Meier methods will be used to compute the survival analyses. The log-rank test and Cox regression analysis will be used to compare the prognosis among patients in the HGS and AG groups. Statistical analyses will be performed using SPSS® Statistics (version 25.0; IBM Corp., Armonk, NY, USA). A two-tailed distribution will be used, and statistical significance will be considered when *P* < 0.05.

#### Monitoring

An independent group of statisticians and physicians will constitute the monitoring committee and will perform visit monitoring on a semi-annual basis. None will have direct involvement in the conduct of this study, nor financial, nor professional interests. The monitoring committee will collect information on the status of accumulation, inclusion/exclusion criteria, and serious adverse events and will strive to provide feedback to participating institutions for early resolution if there are any problems. At the end of the trial, a meeting will be hosted by the participating centres and the monitoring committee to evaluate the final data.

#### Publication of results

We plan to publish the results of this study in a peer-reviewed medical journal, should the results be of sufficient scientific interest.

## Discussion

The choice of drainage approach depends on a patient’s individual anatomical structure, underlying disease, and location of biliary stricture. Both HGS and AG are applicable to patients undergoing malignant biliary obstruction with unreachable papillae. Efficiency and safety are important for such patients, because it is difficult for them to tolerate several procedures and postoperative complications. For these reasons, it is important to develop strategies that would permit them to choose EUS-HGS or EUS-AG, weighing both efficiency and safety.

Several studies have reported the efficiency and safety of EUS-HGS and EUS-AG. Artifon et al. compared the safety and efficiency of EUS-HGS and ultrasound-guided choledochoduodenostomy (EUS-CDS) in a randomised controlled trial, and reported that the technical success rate, clinical success rate, and incidence of complications of HGS were 96%, 91%, and 20%, respectively [18]. Uemura et al. systematically searched the literature up to April 8, 2017, and compared the technical success rates, clinical success rates, and incidences of complications of EUS-CDS and EUS-HGS in systematic evaluation and meta-analysis. The technical success rates, clinical success rates, and incidences of complications were 93.7%, 84.5%, and 18.8%, respectively [19]. In a prospective cohort study, Do Hyun et al. found that the success rate of EUS-HGS was 89%, the incidence of complications was 12.5%, and the success rate of EUS-AG was 57%. In their study, no patient in the EUS-AG group experienced postoperative complications [17]. Iwashita et al. reported that the overall success rate and incidence of complications of EUS-AG were 77% and 5%, respectively, by directly adding the number of cases reported in each searched study [23]. Contrary to other previous reports, Ardengh et al. reported that the success rates of EUS-HGS and EUS-AG were 83.3% and 100%, respectively, by reviewing the different EUS-BD approaches in two hospitals [11]. According to most current studies, the success and complication rates of EUS-AG are both lower than those of EUS-HGS. This may be because EUS-AG is more complex and no permanent fistulas or changes of anatomical structure are created [9]. Nevertheless, the stent in EUS-AG is placed through the tumour, and the growth of the tumour is the main cause of stent re-obstruction. Because a perforating fistula is absent, another intervention for postoperative obstruction will be more difficult in EUS-AG than in EUS-HGS [24]. EUS-HGS may result in more pneumoperitoneum and bile leaks because the fistula traverses the peritoneum [12]. Intraperitoneal deployment is also a potential adverse event of EUS-HGS, which can be fatal [25, 26].

To date, all reports of the safety and efficiency of EUS-HGS and EUS-AG were based on observational studies and randomised controlled studies with another technology or evidence-based medicine. Ardengh et al.’s contradictory findings also appeal to the need for a further comparative study of EUS-HGS and EUS-AG. To our knowledge, ours will be the first study to compare these two EUS-BD approaches directly in a multicentre, randomised, controlled trial. The clinical economic indexes will also be compared, as they may also affect the patient’s choice.

There are limitations to the present study design. The internal biliary drainage operation is a palliative treatment for unresectable malignant biliary obstruction. The lifetime of patients included is limited, because death caused by cancer progression is inevitable. Therefore, the long patency of the stent is difficult to evaluate.

## Trial status

The protocol version number is Ver 1.4, which was registered on 15 January 2019 (ChiCTR1900020737). Patient enrolment will begin on 1 August 2020, and completion is expected by 31 August 2022.

## Supplementary information

**Additional file 1.** CONSORT 2010 checklist of information to include when reporting a randomised trial

**Additional file 2.** SPIRIT 2013 Checklist: Recommended items to address in a clinical trial protocol and related documents.

## Data Availability

The datasets used and/or analysed during the current study are available from the corresponding author on reasonable request.

## Abbreviations

ERCP: Endoscopic retrograde cholangiopancreatography
EUS-BD: Ultrasound-guided biliary drainage
IH: Intrahepatic
RV: Rendezvous technique
EUS-HGS: Endoscopic ultrasound-guided hepaticogastrostomy
EUS-AG: Endoscopic ultrasound-guided antegrade technique
HGS: Hepaticogastrostomy
AG: Antegrade technique
ChiCTR: Chinese Clinical Trial Registry
CT: Computed tomography
FAS: Full analysis set
EUS-CDS: Endoscopic ultrasound-guided choledochoduodenostomy

## References

[1] Wang K, Zhu J, Xing L, Wang Y, Jin Z, Li Z (2016). Assessment of efficacy and safety of EUS-guided biliary drainage: a systematic review. Gastrointest Endosc.

[2] Jovani M, Ichkhanian Y, Vosoughi K, Khashab M (2019). EUS-guided biliary drainage for postsurgical anatomy. Endoscopic Ultrasound..

[3] Hatamaru K, Kitano M (2019). EUS-guided biliary drainage for difficult cannulation. Endoscopic Ultrasound..

[4] Khashab MA, Valeshabad AK, Afghani E, Singh VK, Kumbhari V, Messallam A (2014). A comparative evaluation of EUS-guided biliary drainage and percutaneous drainage in patients with distal malignant biliary obstruction and failed ERCP. Dig Dis Sci.

[5] Giovannini M (2019). EUS-guided hepaticogastrostomy. Endoscopic Ultrasound..

[6] Braden B, Gupta V, Dietrich C (2019). Therapeutic EUS: new tools, new devices, new applications. Endoscopic Ultrasound..

[7] Poincloux L, Rouquette O, Buc E, Privat J, Pezet D, Dapoigny M (2015). Endoscopic ultrasound-guided biliary drainage after failed ERCP: cumulative experience of 101 procedures at a single center. Endoscopy..

[8] Khashab MA, Levy MJ, Itoi T, Artifon EL (2015). EUS-guided biliary drainage. Gastrointest Endosc.

[9] Minaga K, Kitano M (2018). Recent advances in endoscopic ultrasound-guided biliary drainage. Dig Endosc.

[10] Tyberg A, Desai A, Kumta N, Brown E, Gaidhane M, Sharaiha R (2016). EUS-guided biliary drainage after failed ERCP: a novel algorithm individualized based on patient anatomy. Gastrointest Endosc.

[11] Ardengh JC, Lopes CV, Kemp R, Dos JS (2018). Different options of endosonography-guided biliary drainage after endoscopic retrograde cholangio-pancreatography failure. World J Gastrointest Endosc.

[12] Kawakubo K, Kawakami H, Kuwatani M, Haba S, Kawahata S, Abe Y (2015). Recent advances in endoscopic ultrasonography-guided biliary interventions. World J Gastroenterol.

[13] Isayama H, Nakai Y, Itoi T, Yasuda I, Kawakami H, Ryozawa S (2019). Clinical practice guidelines for safe performance of endoscopic ultrasound/ultrasonography-guided biliary drainage: 2018. J Hepato-biliary-pancreatic Sci.

[14] Guo J, Giovannini M, Sahai A, Saftoiu A, Dietrich C, Santo E (2018). A multi-institution consensus on how to perform EUS-guided biliary drainage for malignant biliary obstruction. Endoscopic Ultrasound.

[15] Kahaleh M, Artifon E, Perez-Miranda M, Gaidhane M, Rondon C, Freeman M (2019). EUS-guided drainage: summary of therapeutic EUS consortium meeting. Endoscopic ultrasound.

[16] Cotton P, Eisen G, Aabakken L, Baron T, Hutter M, Jacobson B (2010). A lexicon for endoscopic adverse events: report of an ASGE workshop. Gastrointest Endosc.

[17] Do Hyun P, Seung Uk J, Byung Uk L, Soo LS, Dong-Wan S, Sung Koo L (2013). Prospective evaluation of a treatment algorithm with enhanced guidewire manipulation protocol for EUS-guided biliary drainage after failed ERCP (with video). Gastrointest Endosc.

[18] Artifon EL, Marson FP, Gaidhane M, Kahaleh M, Otoch JP (2015). Hepaticogastrostomy or choledochoduodenostomy for distal malignant biliary obstruction after failed ERCP: is there any difference?. Gastrointest Endosc.

[19] Uemura RS, Khan MA, Otoch JP, Kahaleh M, Montero EF, Artifon ELA (2018). EUS-guided choledochoduodenostomy versus hepaticogastrostomy: a systematic review and meta-analysis. J Clin Gastroenterol.

[20] Paik W, Park D (2019). Outcomes and limitations: EUS-guided hepaticogastrostomy. Endoscopic Ultrasound..

[21] Iwashita T, Nakai Y, Hara K, Isayama H, Itoi T, Park D (2016). Endoscopic ultrasound-guided antegrade treatment of bile duct stone in patients with surgically altered anatomy: a multicenter retrospective cohort study. J Hepatobiliary Pancreat Sci.

[22] Mukai S, Itoi T (2019). EUS-guided antegrade procedures. Endoscopic Ultrasound..

[23] Iwashita T, Doi S, Yasuda I (2014). Endoscopic ultrasound-guided biliary drainage: a review. Clin J Gastroenterol.

[24] Ogura T, Kitano M, Takenaka M, Okuda A, Minaga K, Yamao K (2018). Multicenter prospective evaluation study of endoscopic ultrasound-guided hepaticogastrostomy combined with antegrade stenting (with video). Dig Endosc.

[25] Martins FP, Rossini LG, Ferrari AP (2010). Migration of a covered metallic stent following endoscopic ultrasound-guided hepaticogastrostomy: fatal complication. Endoscopy.

[26] Minaga K, Kitano M, Yamashita Y, Nakatani Y, Kudo M (2017). Stent migration into the abdominal cavity after EUS-guided hepaticogastrostomy. Gastrointest Endosc.

